# Canvas Painting Analysis Using Spectroscopic Analysis and Microcharacterisation Techniques

**DOI:** 10.3390/s22041442

**Published:** 2022-02-13

**Authors:** Braeden Borg, Michelle Dunn, Andrew S. M. Ang, Carl Villis

**Affiliations:** 1 School of Science, Computing and Engineering Technologies, Swinburne University of Technology, Hawthorn, VIC 3123, Australia; braeden.borg.96@gmail.com; 2 School of Engineering, Swinburne University of Technology, Hawthorn, VIC 3123, Australia; aang@swin.edu.au; 3 Paintings Conservation, National Gallery of Victoria, Melbourne, VIC 3000, Australia; carl.villis@ngv.vic.gov.au

**Keywords:** cultural heritage conservation, reflectance spectrophotometry, Raman spectroscopy, 3-d optical profilometry, micro characterisation, spatial mapping

## Abstract

Raman spectroscopy is a well-recognised tool for the analysis of materials in canvas paintings. However, it can be difficult to interpret the peaks of the spectra without the additional context of the artwork such as the age, provenance, or colour. Reflectance spectrophotometry can be used to capture the colour of pigments, dyes, and lacquers, but is seldom used to complement Raman data. Additionally, reflectance spectrophotometry results can be influenced by the surface profile of the painting. To overcome these limitations, this work brings together three different analysis modalities to provide a singular, analytical map of the artwork. Raman spectroscopy was used to conduct the chemical identification of pigments, binding media, and varnish present in a synthetic painting sample. Reflectance spectrophotometry was applied to obtain colour information of the surface paint of the sample. Three-dimensional optical profilometry data was used to characterise the micro topology of the paint surface. These three data sets were spatially matched allowing the recorded spectroscopic data to be displayed with the corresponding colour and surface topography across the paint surface.

## 1. Introduction

The conservation of artwork has advanced significantly throughout the past century with the development of innovative and revolutionary technologies. Technologies such as digital spectrometers [1,2], confocal microscopes [3,4], and improved computer software has expanded our knowledge of the chemical makeup of constituents, such as pigments, binding media, and varnishes for paintings. The characterisation of these constituents can assist expert conservators and historians to determine the optimal approach for repairing a damaged piece of art or ensuring its preservation. While the types of technologies that are available for artwork analysis are vast [5] and researchers use multiple techniques when analysing an artwork [6,7,8,9,10], there is limited evidence of these technologies being used to complement each other through data fusion or combined into a singular material information map. The advantages to gain from combining data from multiple sources can be seen in Daffara et al. [11]. They combined data from speckle interferometry and speckle photography to generate maps showing out-of-plane and in-plane displacements. 

The fusion of different data modalities introduces a new and comprehensive method of analysing works of art. However, the integration of these different data types and technologies used to obtain the information initially can be challenging. It is believed that this lack of data conjunction is partially a consequence of analytical software not being designed to make this fusion widely accessible. Moreover, those who will be using this software will be expert art conservators and historians who want the best experience when using the collective data for their analysis. It is for these reasons that the aim of this paper is to demonstrate how data types from different scientific analyses can be combined into one map. The platform in which this data is formed is through a graphical interface that provides a useful experience for experts in the field of art conservation. The way in which spectroscopic, spectrocolourmetric, and morphological data is combined in this interface sets a standard for artwork conservation software. In our work, three state-of-the-art technologies were used to inspect ad hoc canvas paintings. Data obtained from these technologies were combined into an *Artwork Analysis Tool*. This allows artwork scholars to easily extract relevant data from multiple technological sources related to certain points in the image and thereby make informed decisions. Data pertaining to the pigments, binding media, and varnish present in a painting will be acquired with spectroscopic techniques of Raman spectroscopy and reflectance spectrophotometry and relevant databases of spectra. Topological data obtained via optical profilometry compliments this data, permitting a comprehensive micro characterisation of painting surfaces.

Numerous analytical techniques have been used in the literature to identify paint constituents [12,13,14], surface topology [15,16,17], and internal paint structures present in oeuvres (paintings) [18,19]. In this work, the paint constituents present in a painting sample were characterised with spectroscopic techniques. These techniques allowed the chemical structures and associated colours of the surface paint stratum to be identified. Chemical analysis was accomplished with Raman spectroscopy which accurately detects the molecular system excited with infrared light. To determine the colour of the surface paint, reflectance spectrophotometry was used. This data was in turn complemented by topological or surface roughness data from optical profilometry. To the best of our knowledge, the combination of Raman spectroscopy, reflectance spectrophotometry, and optical profilometry has never been accomplished before in the literature for artwork conservation. A custom data structure characterising chemical signatures pertaining to contemporary pigments, binding media, and varnish constituents of a painting was created. Colour data obtained through reflectance spectrophotometry was added to provide context to the pigment chemical signatures and to provide complementary identification through spectral databases. Finally, optical profilometry data was included in the model providing a more holistic view of the surface of the painting sample. 

With the expert knowledge of an artwork conservator, a tool that combines different spectroscopic and profile techniques will help identify painting materials in a comprehensive manner. Data regarding paint pigments, binding media, and varnish measured through experimental procedures can be mapped to their scanned positions of the painting. Any inconsistencies in the expected results for a particular region might correlate to damaged or defective regions in the surface paint layers. Regions that are identifiably damaged by the conservator can be correlated with the high-fidelity spectroscopic data in the graphical map of the combined data. This can help identify which materials need to be used or considered when restoring that region of the painting. The formulation of a digital conservational tool for expert conservators and historians is an important endeavour that aims to help preserve the cultural heritage and history of human expression.

### Background

Raman spectroscopy is a powerful tool in the identification of chemical structures or signatures in various materials. As a result of its precision and non-destructive nature, Raman spectroscopy is an established tool for artwork analysis [20]. Raman spectroscopic instruments can probe underneath the primary paint layers of a painting and make subsurface measurements. Raman spectroscopy detects the photon interactions between a molecular system and excitation light source. This phenomenon alters the measurable energy across the entire system. Different molecular systems given the same light excitation conditions will have characteristic energy changes. After a collision has occurred between the photon and the molecule, the energy of the emitted photon will either increase or decrease (anti-Stokes and Stokes Raman scatter respectively) depending upon the ambient state of the molecular system. This energy differential can be measured with great accuracy to determine the type of chemical probed.

Raman spectroscopic instruments can probe underneath the primary paint layers of a painting and make subsurface measurements. Most commercial Raman spectrometers operate through a confocal lens, making the entire process scan specimens at the micrometre scale. This provides a powerful tool that can conduct micro characterisation of painting surfaces. Raman spectroscopy can be enhanced at even higher magnifications with the integration of surface plasmonic enhancement. Thus, Raman spectroscopy has proven high potential for precise and powerful analysis of chemicals present in the constituents of paintings. 

Spectrophotometry is also commonly employed to analyse artworks such as paintings. Among other applications, it allows the analysis of how the fabric beneath the paint can absorb the various chromatic components [21] and the identification of rare dyes and pigments when used in combination with other analysis modalities [22]. Spectrophotometry analyses the nature of visible light. More specifically, it analyses how light has interacted with a material surface and provides information about the perceivable colour. Reflectance or fluorescence spectrophotometry captures remitted light through its optical configurations and focuses it onto a monochromator to generate a spectrograph. This process splits the captured beam of light with a fine grating plate into its different wavelength components for examination. The grating plate is a thin, metallic disc with a highly refined sawtooth-patterned surface that diffracts different wavelengths at each sawtooth peak. The diffracted light can then be measured with photosensitive detectors and processed with mathematics to generate a spectrum of the captured light. The spectrum generated is highly characteristic of the colour scanned, and as such describes the measured sensitivity of remitted radiation with a corresponding wavelength. Alterations to the surface, such as discolouration due to natural degradation processes, will also influence the measured colour. Hence, spectrophotometry exhibits great potential for accurate colour analysis of painting surfaces that could be useful for conservators when determining the pigments and, indirectly, the varnish present in an oeuvre. 

While Raman spectroscopy and spectrophotometry analyse the composition of the materials used to make an artwork, three-dimensional (3-D) optical profilometry is used to conduct investigations of the surface condition for conservational purposes. Recently it has been used to monitor the cleaning process [23] and explored for potential use in the printing of a replica artwork [24,25] allowing for tactile exploration. Interferometry forms the basis of many optical profiler tools and relies upon the interference patterns of light reflected from a surface. In Michelson interferometry, a commonly used method in optical profilers, the light beam is split and travels different paths to the detector. As each beam is of the same frequency, when the light waves recombine, an intensity pattern is produced that is caused by the phase difference between them. Analysing this phase difference provides information about the difference in distance travelled by each beam of light. This can be configured to detect the height or depth of the surface at the micrometre scale. Optical profilometers are capable of automatically scanning across a surface and hence building up a three-dimensional surface profile of the sample.

All of these different methods of obtaining appropriate and pertinent data are similar in their spatial relation with canvas paintings. Spectroscopic information can be correlated with different regions of paint visible to the eye, or additional painting constituents located deeper within the subsurface layers. Morphological information can also be associated with regions that showcase painting stylometry or regions with physical damage. As such, the spatial correlation of these data modalities was identified to be an important component of the *Artwork Analysis Tool*. However, as highlighted previously, the combination of data from different analytical modalities can be challenging. A Raman microscope provides very accurate information at the micrometre scale whereas a handheld spectrophotometer provides colour detail at the millimetre scale. The benefit of using a digital platform to combine this data together is that it can be aligned and restricted as needed to best suit the user. As such, information will be lost from the Raman and micro profiler data at a spatial level, but the quality of those fewer regions will improve when complemented with the spectrophotometric data. This approach aims to provide the user with the information they can use practically.

## 2. Materials and Methods

### 2.1. Sample Preparation

**Figure 1:**
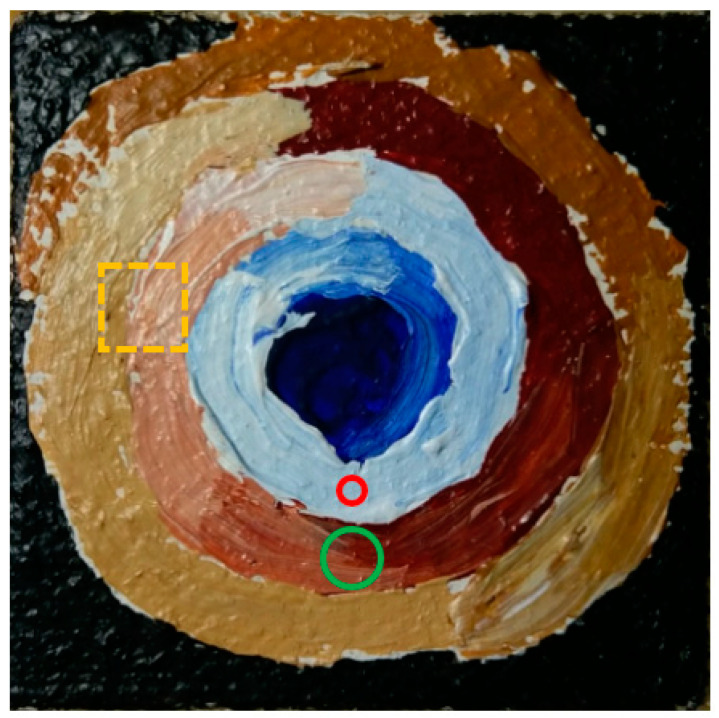
The sample painting used for this study. The individual points analysed by Raman spectroscopy (**red**) and reflectance spectroscopy (**green**) are highlighted. The region scanned using the 3-D optical profiler is outlined in orange.

For this study, a painting sample was created by an art conservator from the National Gallery of Victoria (NGV). The origin of the painting materials used was not known in detail as the analysis focused on identifying these constituents experimentally and later discussing the findings with the art conservator. The sample had a surface area of 50 mm by 50 mm and an average depth of 6 mm from the surface paint layer. Some rudimentary details on the materials used in the painting sample were provided by the art conservator. Canvas fabric was wrapped around a thin block of aluminium and bound to the surface with an adhesive. An adhesive primer and white paint layer were then applied. Two coats of paint composed of mineral pigments with a synthetic binding media were then added to each painting. No varnishes or lacquers were present in the sample painting so that the paint surface could be analysed reliably. The sample, shown in Figure 1, was composed of several warm colours transitioning throughout the spiral pattern from the centre. 

The preliminary paint layers on the canvas were relatively thick and illustrated an undulating topology at the micrometre scale. The surface irregularity is an important consideration as the spectrophotometer device must not touch or interfere with the sample surface during operation. In addition, ambient light conditions have an impact on the precision of measuring the colour properties of the painting surface. If the surface is too rough, then the performance of the spectrophotometer can be affected. 

### 2.2. Raman Spectroscopy

Raman spectra of the painting sample were obtained using an inVia™ Confocal Raman Microscope (Renishaw, Wotton-under-Edge, Gloucestershire, UK). Raman analyses of the painting sample were achieved at the micrometre scale with the use of the confocal lens microscope, allowing micro characterisation of the painting surfaces. The platform that the sample was mounted on was motorised and allowed for spatial scanning (referred to as a raster scan) of the sample. To ensure consistent analysis with the microscopy set-up, a custom fixture was designed for the painting sample. The specialised fixture ensured that the sample did not shift or vibrate during scanning to improve the quality of the experimental data. It had an imprinted reference point in one of the corners that was used as a datum, keeping the Raman analysis consistent between scans and allowing spatial matching with other data types.

**Figure 2:**
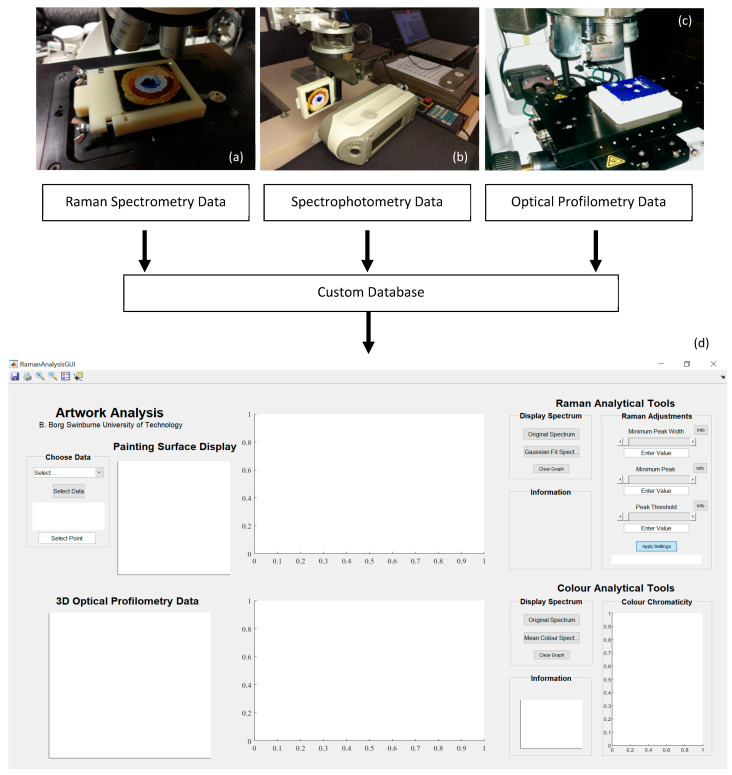
Schematic of the data acquisition feeding into the custom database and graphical user interface (GUI). (**a**) Raman Spectroscopy with the white laser highlighting the position of the reference datum (**b**) Spectrophotometer with the sample held in the end-effector of the robot (**c**) 3-D Optical Profilometry, with the sample mounted beneath the adjustable lens turret and (**d**) the MATLAB GUI illustrating the display with Raman and colour data loaded into the platform and graphed for analysis.

A 785 nm (λ_0_) near-infrared diode laser directed through a 5× objective lens was used to scan the painting surfaces. The aperture and focal length were set to 65 m and 80 m respectively for microscopic observation. All spatial positions were measured from the reference datum embedded in the fixture. An example of the experimental setup is displayed in Figure 2a, with a white laser highlighting the position of the reference datum. Four spatial scans with equal area resulting in quadrants were completed for each painting specimen in a raster pattern. Each scan sampled the surface at intervals of 5 mm across each raster row resulting in a total of 144 data points. Finer resolution is possible with a spatial accuracy of up to 1 µm. However, since the total time required to scan the 144 data points was 1.3 h per quadrant, this resolution was reserved in the interest of time. 

Raman spectra were obtained at each node with a ten-second exposure on the painting sample within the 200 to 2000 cm^−1^ Raman shift domain. This range was specified because of its sensitivity in detecting chemical structures in paint constituents. A 1200 L/mm (633/780) diffraction grating was used to measure the Stokes Raman scattering. The laser was operated at 10% of its maximum power, ensuring that the specimen was not overexposed and damaged by the laser source during scanning. Preliminary results showed that a high-power setting was not necessary to gather enough Raman counts over the ten second scanning period. The total scanning time of each node was typically around 19 s, as it included the post-processing time of the Renishaw software after accumulation. Finally, the motorised stage needed to move after each scan to complete the raster pattern adding time to the entire process.

**Figure 3:**
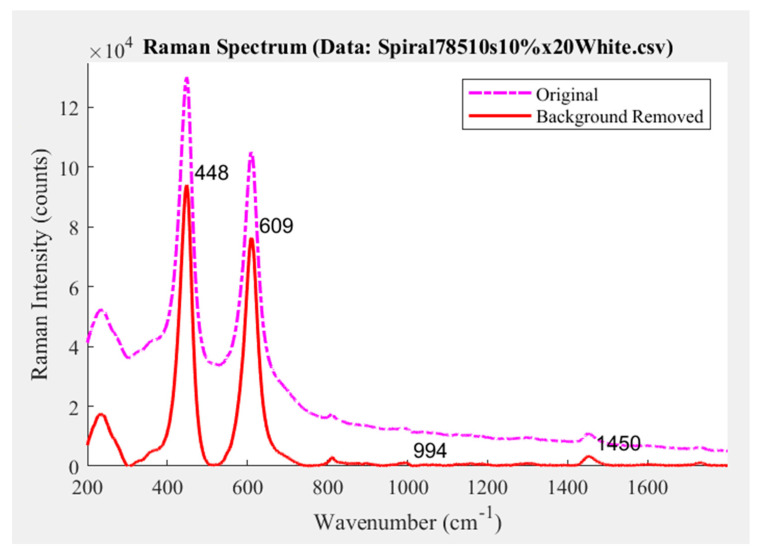
Raman spectra example from the sample painting displaying original and processed spectral data with Raman peaks at 233 m, 448 s, 609 s, 813 w, and 1450 w cm^−1^. The processed spectrum in red has had noise smoothed out of the data and its fluorescence background removed.

Before analysis, the Raman data was post-processed using two algorithms. The first algorithm removed the background fluorescence from the data and the second filtered out measurement noise in the spectrum. Background fluorescence that was predominant in the spectrum was removed using an algorithm produced by Cadusch et al. [26]. Measurement noise in the spectrum was then reduced using a second-order Savitzky–Golay filter with a square mask size of nine pixels. It was found that a second-order Savitzky–Golay filter performed the best in interpolating the data when a higher magnification objective was used. An example of a typical Raman spectrum and the result of the spectral processing is shown in Figure 3. This data comes from the pale blue region near the centre of the image and is highlighted with a red circle in Figure 1.

### 2.3. Reflectance Spectrophotometry

Reflectance spectrophotometry was obtained using a Spectrophotometer CM-2600d (Konica Minolta, Tokyo, Japan). The spectrophotometer measures reflectance at the surface of an object and generates an optical spectrum. For one measurement, the device obtained a spectrum with specular components included (SCI) and specular components excluded (SCE), improving its range over different surfaces. The two main factors that influence differences between the SCI and SCE components are surface topology and diffusivity. If the surface is highly irregular or diffuse, then the resulting measurements will appear to be less saturated and duller in colour. Due to the diffusivity of the surface layer on a canvas painting, the perceived colours are expected to be less saturated. SCI is also generally used to measure the colour of surface material, while SCE is used to measure the apparent colour of the surface as determined by inferred surface diffusivity and glossiness. 

The spectrophotometer can be operated with diverse apertures at the scanning module interface. For this analysis, the spectrophotometer was used with an aperture plate of 3 mm in diameter. As such the different possible colours present within the aperture circle were averaged across the measured reflectance spectrum. Spectra acquired from the spectrophotometer were in the wavelength range of 360 nm to 740 nm with a surface reflectance range of 0 to 175% (up to 2.43 dB) of measured reflectance (as per the device specifications). The standard illuminant setting of D65 (daylight) and observer of 2° was selected. The colour, because of the selected observer, provided intrinsic colour data in the CIE 1931 (International Commission on Illumination) space. As will be discussed in the digital interpretation of the colourimetric data, the colour information obtained from the spectrophotometer was represented as a reflectance spectrum and additionally in the CIE 1931 colour space. This enabled the intrinsic colour to be visualised as it would naturally be perceived by a human eye without irregular influences. The spectral range of the spectrophotometer demonstrated that only colours from the visible spectrum were measured. Spectral acquisition of both SCI and SCE components successively was measured within four seconds for each scan. 

Since the spectrophotometer CM-2600d is a handheld device, a novel approach to making the scanning process autonomous was developed. A SCORA-ER14 (Intelitek, NH, USA) robotic arm was used to generate a raster scan of the painting sample to acquire the colour spectra. The scan pattern was programmed to be synonymous with the Raman raster scan settings. As such, a point every 5 mm with the given aperture was measured leading to 144 points across the painting sample. The experimental set-up is shown in Figure 2b with the painting sample held by the pneumatic end-effector of the robotic arm. Prior to any scanning, the spectrophotometer was calibrated and zeroed as recommended. The zero calibration was done with the device exposed to an open space and the white calibration used the provided white plate. The orientation of the sample painting during the raster scan is shown in this image. The interface between the spectrophotometer aperture and painting surface needed to be almost touching to ensure optimal scanning quality. A scanning distance of about 1 mm was set. However, given the irregularities of the surface of the sample, adequate space was made in between the specimen surface and the aperture opening during the scanning procedure was not perfectly consistent. Three measurements were obtained at each point and averaged by the device to remove the influences of noise or any irregularities in measurements due to positioning.

Once the spectra have been obtained, the colour can be determined from the spectra. For cultural heritage compounds, in particular, online databases can be utilised to compare the spectra and determine compounds that produce the same or similar spectra, thus complementing other analysis methods such as Raman spectroscopy. Such databases can be found at https://spectradb.ifac.cnr.it/ (accessed on 22 December 2021).

### 2.4. Optical Profilometry

The micro topology of the painting sample surface was captured using a Contour GT-K 3-D Optical Microscope (Bruker, Billerica, MA, USA). The optical profiler had a vertical resolution of less than 0.01 nm and a lateral resolution of 0.38 μm. The maximum scanning rate was 47 μm/sec. The machine applied Michelson interferometry to measure optical distances across the surface of a specimen providing height value data. Vertical scanning interferometry (VSI) was the primary mode used with this equipment when imaging a specimen. In addition, the light within the visible domain of the electromagnetic spectrum was used. The equipment permits white, green, and a narrow green wavelength band as the primary coherent light source. The metrology datasets were achieved by taking many height measurements across an x-y coordinate system. Since all spatial dimensions were obtained from the VSI measurements, 3-D models and graphs could be generated and analysed in the digital software. Taking a gradient map (monochromatic) of any image and processing the pixels spatially also allowed the topology to be analysed. 

The Contour GT-K 3-D Optical Profiler uses a precise, motorised stage to translate the specimen horizontally in-plane (x and y axes). The optical head holds all of the required optics, laser source, and digital sensors to conduct interferometry. The head is also motorised along the z-axis, enabling the device to change its focal length and depth scanning according to the specimen height and objective used. However, once the specimen is positioned underneath the lens, the head remains stationary relying upon the internal optics to accurately adjust for interferometric readings. Once the field is scanned, the motorised stage will translate the specimen so that the next region can be scanned. This movement results in vibration, so a custom base was constructed for the canvas to make it more robust when the hardware was operated.

The 3-D optical profiler was used in the default VSI mode with a 2.5X objective to measure the topology of the painting sample in regions up to 12.5 mm × 25 mm. This method was to ensure that the entire surface topology could be documented as a visual reference, and, of course, correlated with the individual points where Raman and colourimetric spectra were measured. Since these areas were quite large for the surface imaged, smaller rectangular regions typically within 8 mm × 8 mm were acquired for analysis. The profiler was typically configured to scan to a depth of 500 microns, with 100 microns of back-scan. In addition, the wide green bandwidth illumination source was used. It is important to note that larger scan depths will lead to longer scan durations. As such, the scanning depth and back-scan parameters should be set proportionally to the undulation of the paint surface and maximised only if damaged areas are visible. Moreover, in the case where a much larger work of art is analysed, it is recommended that only the regions of interest (likely overlapping with the spectral-spatial data) are characterised. 

### 2.5. Artwork Analysis Tool

Data obtained from Raman spectroscopy, spectrophotometry, and the 3-D optical profiler were assembled and analysed in MATLAB (The MathWorks, Inc., Natick, MA, USA). A graphical user interface (GUI) was developed in MATLAB as an analytical tool to interpret the Raman and colour spectral datasets for the painting sample, as well as the surface topology. The interface allows the user to load data into the platform and conduct analyses on the painting. Spectra from both the Raman and spectrophotometer techniques can be graphed simultaneously, giving the user the ability to analyse and compare the complementary data at a corresponding point on the painting surface. In its current form, the tool only allows for disparate points to be analysed at any one time. Nevertheless, the surface 3-D profile data can provide detail across a wider area with a resolution suitable for micro characterisation. An image showing the position of the spectroscopic and surface micro topological data sources in the MATLAB GUI is displayed in Figure 2.

The GUI was developed to represent the final spatial set of spectroscopic data and the surface topology images. A MATLAB script was written to post-process the spectral datasets prior to being loaded into the GUI. The GUI would then provide the user with the ability to inspect the data across the surface of the painting being analysed. To correctly condition the Raman spectra, the noise and background fluorescence signal was removed. The Raman spectrum of a point on the sample painting when loaded into the GUI had its background noise removed and the excitation peaks of interest were highlighted. For the same point, the processed data obtained from the spectrophotometer was displayed. The raw SCI and SCE spectra were plotted, with the corresponding CIE 1931 colour space representation and chromaticity chart point. The MATLAB script converted the raw colourimetric and spectral data from the device to represent the data accordingly. Finally, the surface topology data were shown for that point. In this case, since the painting sample was sufficiently small, the colour contrast map of the topology (colours representing depth and back-scan) for any point was attributed to a larger quadrant imaged. Typically, important details in the surface 3-D profile are evident over larger areas where brush stroke patterns or damaged paint material can be seen. An advantage of the GUI being programmed in MATLAB is that there is an opportunity to customise the digital platform for the types of data being fused together.

## 3. Results

### 3.1. Raman Data Analysis

In the *Raman Analytical Tools* section of the *Artwork Analysis Tool*, Gaussian functions are used to highlight where major peaks exist in the Raman spectra. The parameters that determine the position of peaks in the data can be changed dynamically while the data is displayed. The shape characteristics of the fitted curves aim to reflect the nature of the chemical signature being highlighted. As such, the Gaussian fitting algorithm detects attributes of the spectral peaks such as their position, width, and peak prominence (how much a peak stands out from the surrounding baseline). Due to the symmetric nature of Gaussian curves, more complex peaks will not necessarily be characterised as thoroughly. As such, characteristics such as shoulder (or companion) peaks and the breadth of major peaks will not be highlighted by the fitting algorithm. Analysis by eye is necessary to discern these details and it is important to note that these subtle characteristics may not be apparent to the untrained eye.

**Figure 4:**
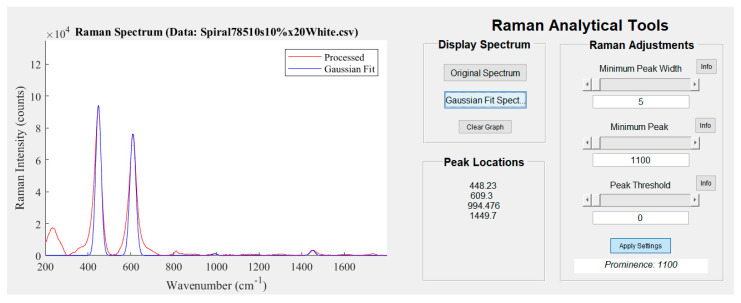
Interpretation of the previous data from Figure 3 showing the detected Raman peaks in the spectrum using the Gaussian fitting tool in the GUI. The Gaussian functions fit the data well and automatically highlights peaks that were previously identified by eye.

Fitting Gaussian curves to the Raman spectra helps the user to correlate them to chemical compounds by comparison with a spectral database. A Raman spectrum obtained from the sample painting is displayed in Figure 4 and the Gaussian functions fitted to this spectrum are apparent in the graphical display. The spectrum showed chemical signatures that are characteristic of white pigments. For each spectrum, the position on the painting sample where it was scanned by the micro-Raman spectroscope was shown in the main GUI as well as the 3-D profile of the surrounding area. By illustrating the region where the painting was scanned, the context of the colour of the paint (or if the painting exhibits any surface damage) is a key insight when finding the compound in a reference library. A damaged surface may expose material underneath the primary surface layers of a painting, thus yielding distinct chemical compositions in the spectrum. 

To demonstrate the kinds of analysis that can be performed, the Raman spectra of the sample painting were obtained from the point shown in Figure 1. The resulting data, provided in Figure 3 (fluorescence background removed and signal smoothed) and Figure 4, displays peaks at 233 m, 448 s, 609 s, 813 w, and 1450 w cm^−1^. The relative intensity for identified Raman peaks are indicated here as; strong (s), medium (m), and weak (w). Similar peaks at 232 m, 447 s, and 609 s cm^−1^ from Burgio et al. [27] were found to correspond with Rutile titanium oxide (TiO_2_), a white pigment. Hence, the white paint layer applied after the primer (as described in Section 2.1) was detected and identified as Rutile. The minor peak at 812 w cm^−1^ likely correlated to the presence of *Perspex* polymethyl methacrylate as a synthetic binding agent in the paint. Given that the paintings were produced with contemporary materials, the paint was likely prepared with a synthetic binder. Additional analysis of the spectra showed that there were shoulder peaks at 356 w cm^−1^ and 270 w cm^−1^. These Raman peaks may pertain to underling materials in the paint structure which were detected at relatively lower intensities. The peaks were, nevertheless, contributing to the previously identified peaks in the spectra. It was expected that these chemical structures either complemented the pigments in the perceived colour of the paint or were fundamental chemical components to the Rutile titanium oxide white pigment.

The smaller peak highlighted in the spectra at 1450 cm^−1^ depicted the minor presence of another material. After comparing the peak location with the spectral library, it was found to be challenging to identify which pigment or media was discovered. There are a few materials with similar sensitivities to this wavenumber, but the 1450 cm^−1^ region is typical for organic compounds, and we knew that the paint sample was made using synthetic compounds. It was possible that some external compound may have been introduced accidentally into the paint mixture. During the preparation of the paint, particulates from the environment or residue material on the tools can make their way into the new paint mixture. It was also possible that the sample painting was contaminated during transportation or in the laboratory. Due to the small number of Raman counts measured for this peak, it was feasible that the region scanned had a defect in the painting surface. Additional analysis of the red and yellow pigments present in the painting could provide insight into unknown chemistries. 

It is important to note that the peaks identified may signify the presence of a painting compound not yet described in the literature. Referencing materials by their chemical signatures to a reference library or set of libraries can be challenging. As such, any form of complementary data can help in narrowing the possible chemical compounds that are present in a painted artwork. The potential of Raman spectroscopy for reliable and accurate identification of painting constituents is demonstrated by the fusion of complementary data maps. It is for this reason that spectrophotometry was employed to obtain clarity on the chemical signature of painting constituents.

### 3.2. Colour Data Analysis

The colour analysis component of the *Artwork Analytical Tool* provides a graphical representation of the reflectance spectrum and a visualisation of the colour. Both the SCI and SCE components of the reflectance spectrophotometry data are displayed. These two components are generally similar in both profile and magnitude but can provide key details into the painting surface. The colour data recorded by the spectrophotometer is based on the CIELAB colour space (also referred to as L*a*b*). It encodes perceptual brightness (L*), red-green (*a), and yellow-blue (*b) colour series. For this study, data in the L*a*b* colour space were converted into red, green, and blue (RGB) components. While this conversion maintained the fundamental colour of the measured sample, it lost information on the lightness or diffusivity of the actual paint surface scanned. 

To correctly display the nature of colour, a 3-D graph of the L*a*b* colour space (due to the complex topology) needs to be produced. However, it was determined that this depiction of the colour information was not an intuitive way to visualise it in the *Artwork Analytical Tool*. As an alternative, three displays of the colour data reduced in dimension were produced. The first two were related to the SCE and SCI components of the reflectance spectra converted into an RGB value. The third representation plotted the colour value on a chromaticity chart, which was a two-dimensional (2-D) space for displaying the L*a*b* colour parameters. Although some higher dimensional information was lost in these versions of the data, they still conveyed important information on the colour measured from the painting surface. To display the colour on a chromaticity chart, the original L*a*b* colour space data was converted into the *CIE 1931 xyz* colour space (derived from XYZ through elementary relationships) with the *z* dimension omitted. The *x* and *y* components could then be plotted directly onto the chart. The 2-D chromaticity chart in the GUI represents the corresponding extended gamut colour for both the SCI and SCE components of the spectrophotometry data. Once the spectra are obtained, the user can then either evaluate the colour shown in the *Information* panel of this section of the *Artwork Analysis Toolbox* or can compare the spectra directly to a reflectance spectroscopy database such as that produced by Picollo et al. [28].

**Figure 5:**
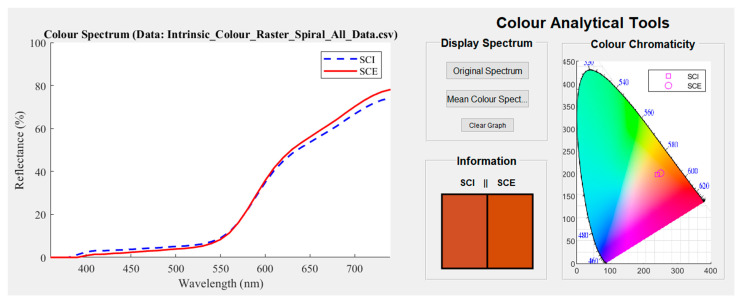
Original spectra of the previous data where the specular components included (SCI) and specular components excluded (SCE) components for the measurement position are illustrated. The perceived and intrinsic colour is also provided under the information section, with the chromaticity position displayed on the far right.

To demonstrate how the colourimetric data is analysed, original spectra of the sample painting as shown in Figure 1 (with the visual representations of the colour in the GUI), are provided in Figure 5. This set of reflectance spectra shows a broad range of high reflectance at wavelengths from 550 nm to 740 nm. This large area of reflectance included yellow, orange, and red colours in the visible spectrum. The colour visualisations of the two spectral components displayed much more clearly a burnt-orange, brown colour. For colours that are highly desaturated or high in shade, one should refer to the colour that is visible in the marker on the digital photograph of the painting. The region, as shown in the image, had a mixture of brown colours from chestnut to burnt-orange. Smaller patches of light blue may have impinged upon the aperture from the centre of the painting. The SCI and SCE components of the spectra graphed in the GUI indicated that all these colours could have been captured in the aperture simultaneously. The acquired spectrum shown in Figure 5 helped to identify that the colour was a burnt-orange. This matched well with the spectral profile which could be attributed to red ochre, a pigment constituted by the hematite mineral (α-FeO) with silica and clay. The red hue was due to the ferric oxide and the spectrum could vary depending on the type of binding media and the hematite content, but its shape remained well recognisable as earth containing the hematite chromophore. This demonstrated that reflectance spectroscopy is a useful tool in identifying paint colour by an analysis of the reflectance spectrum and digital colour values. It provided information that was not only useful for identifying paint colour but was also complementary to Raman spectroscopy for more precise micro characterisation.

The surface diffusivity, or the topological roughness of the surface paint layer, plays an important part in colour detection. For several positions measured on the sample surface, the paint thickness and topology changed by a recognisable amount. A rough surface can alter the performance of the spectrophotometer and cause erroneous differences between the SCI and SCE components. It was for this reason that supplementary analysis with a 3-D optical profiler was employed to aid in the high-fidelity visualisation of the surface topography to better understand the reflectance spectra.

### 3.3. Optical Profilometry Data Analysis

**Figure 6:**
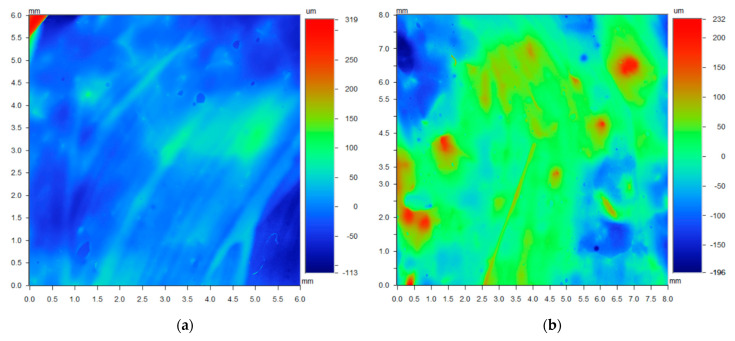
Optical profilometer data: (**a**) Gradient colour map of the surface topography illustrating prominent stylometric characteristics from the brush strokes; (**b**) Gradient colour map of a prominent feature that originally looked like a scratch to the naked eye.

Contour gradient colour maps obtained from the 3-D optical profiler are integrated into the *Artwork Analysis Tool*. The data obtained from the profiler illustrates the topographical features of the region using a gradient scale. The dimensions of each image are dependent on the field of view of the scanner (objective and magnification optics) at the time of examination. Surface roughness parameters can be calculated statistically to describe the surface topography in detail. The images displayed in Figure 6 provide holistic details of a paintbrush forming the primary paint layer. The region shown in the image on the right has its position superimposed on the painting sample in Figure 1. Small defects as a result of dust particles and air bubbles are also a prominent feature in this topographical image. The technique of applying the paint using a brush affects the profile of the paint layers substantially at the micrometre scale. Different styles, weight, and paint volume affect the resulting thickness and topology. The image in Figure 6a, shows the profile of a paint layer moulded by the bristles of a brush. Bristle marks have been imprinted into the surface paint layer with different hefts. Narrow hills and valleys were moulded in the paint when originally applied, signifying the direction of the paint strokes. By determining the brush stylometry of the painter, it may be easier to discover the identity of an unknown original artist.

Figure 6b also shows another region that appeared to have a scratch mark. The considerable amount of height deviations in the 3-D image confirmed that the painting surface was deeply damaged by a sharp tool. Insight from an expert conservator from the NGV verified that the damage was likely caused by a scalpel. The varying applications of paint from the brush were exemplified by the different textures and profiles in the surface profile of paintings. Large valleys in the top left and mountains near the top right were presented on the canvas surface. It is from this information that insight into the provenance and artist heritage of a painting can be deduced. Moreover, the micro topology directly complemented the conclusions that can be drawn from spectroscopic techniques that rely on understanding the material that is being analysed.

## 4. Discussion

**Figure 7:**
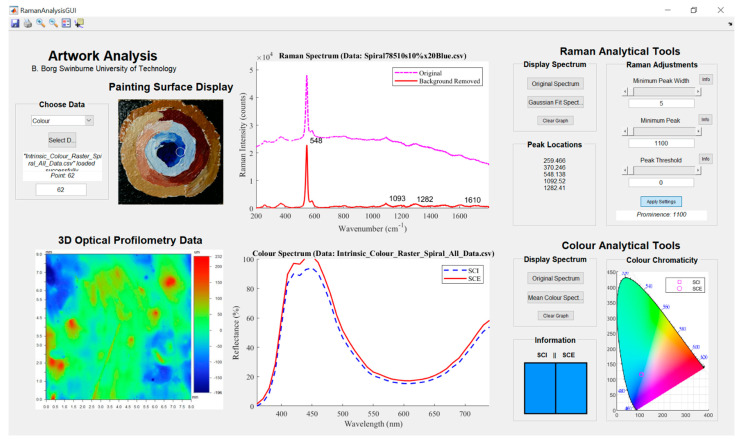
The *Artwork Analysis Tool* displays the analytical results from the three different analysis methods on the light blue paint region near the centre of the oeuvre.

The results discussed previously for each of the analytical techniques demonstrated how such an analysis could be made on a canvas painting. The information obtained from each technique was generally insightful and useful for an art conservator. However, the information when juxtaposed with findings from other techniques was found to provide additional insight and solidify findings that were made about the painting. To further demonstrate this, and with an example derived purely from the *Artwork Analysis Tool*, analysis of the painting sample at the blue section of the spiral was conducted. In this area (as shown in the *Painting Surface Display* section in Figure 7) a corresponding Raman spectrum, reflectance spectrum, colourimetric parameters, and surface metrology data have been displayed for point 62 of the entire surface scan. 

The peaks of the Raman spectra from the point highlighted in the *Painting Surface Display* are 259 w, 370 w, 548 s, 1093 w, and 1282 w cm^−1^. From this set of identified peaks using the Gaussian fitting tool, the only strong and prominent peak is the one at 548 cm^−1^. Using the library from Burgio et al. [27] as a reference, the primary compound was determined to likely be Lazurite in a sodium alumino-silicate matrix. This is used as a synthetic paint pigment called ultramarine blue. If mixed with white paint, then the colour would appear more like that displayed in the *Colour Analytical Tools* section. In a previous analysis of the pale blue paint region from Figure 3, it is known that Rutile was added, a white paint layer added after the primer, and Perspex polymethyl methacrylate was used as a synthetic binding agent in the paint. Given that regions of the canvas substrate are visible in the painting from the *Painting Surface Display*, less than a few paint layers were applied indicating that the primer and additional paint layer could have mixed. Alternatively, the Rutile white paint could have been added to this section of the oeuvre to help form the colour transition in the spiral. The Raman spectrum also indicates a possible minor presence of Phthalocyanine blue, a synthetic pigment at 255 cm^−1^, and Bright red β-naphthol at 1099 and 1282 cm^−1^. Although it would appear unusual to see a red pigment, it is known there are some red wavelengths of light in the paint colour as evidenced by the reflectance spectrum. The weak addition of this red pigment and the white paint differentiates the painting sample surface from a pure Lazurite blue colour. By combining the analyses of the Raman spectra, reflectance spectra, and colourimetric information, important information formed with a variety of perspectives will be most useful for the expert art conservator.

The 3-D optical profilometry data (Figure 6a) reveals that the point being analysed in this instance is in an area spotted with small peaks and paint layer striations. The striations are likely caused by the stroke of a brush when the final paint layer was applied. With the difference between the highest and lowest points in the order of 428 um, there is evidence that the roughness of the surface topology may have an impact on the perceived colour. This is evidenced in the *Information* portion of the *Colour Analytical Tools* in the *Artwork Analysis Tool* (Figure 5) where the SCI and SCE representations differ at the double peaks around 430 nm. It is also important to note that the areas where there are clear circular mounds might be the presence of a material dissimilar to the surrounding paint. These will consequently have an influence on the Raman spectra obtained in those regions leading to erroneous conclusions.

The fusion of three different analytical techniques has been demonstrated with the use of the *Artwork Analysis Tool*. The intrinsic information that enables the identification of the constituents, colour and surface metrology of canvas paintings are all provided in an easy to navigate, visual platform. Analysing works of art using Raman spectroscopy, reflectance spectroscopy, and 3-D optical profilometry have been demonstrated as useful modalities individually in the literature and in this study. However, it has also been exemplified that the combination of these different techniques provides useful insights into the other data. Colour information complements the identification of pigments and binding media in paintings. For the sample painting, Lazurite was identified as a major blue pigment in the centre spiral. Bright red β-naphthol and Rutile white paint were also identified in the spectral signature, confirmed by complementary data from the reflectance spectra and visual representations of the colour. The surface metrology indicated that certain specks of surface paint could introduce outliers into the Raman spectra leading to inaccurate conclusions. Other than the small peaks, the paint surface was relatively uniform and showed no evidence of surface damage that would otherwise become a focal point for an art conservator to investigate. 

## 5. Conclusions

Raman spectroscopy, spectrophotometry, and optical profilometry are all important techniques for the characterisation of the materials and surface characteristics that make up painted works of art. These three state-of-the-art techniques bring different information to the analysis but are usually considered in isolation. Raman spectroscopy and reflectance spectrophotometry exhibit potential in being used together as a means to identify pigments, binding media, and varnish constituents in paintings, while optical profilometry supplements this with surface characteristics providing insight into the spectroscopic data.

To demonstrate the power of these three techniques together, a sample painting was constructed at the NGV and comprehensive data was collected from each in the form of Raman spectroscopy, spectrophotometry, and optical profilometry. A confocal Raman microscope was employed to collect a set of Raman spectra corresponding to different regions on the surface of the painting sample. The collected Raman spectra underwent post-processing to remove unwanted noise and background fluorescence from the data. Colour spectra were obtained autonomously with the use of a reflectance spectrophotometer and robotic arm, translating the painting sample across the aperture of the handheld device. A spatial set of data relating colour to different regions of the painting surfaces was constructed in this way. The resulting spectra can be matched against spectra databases available online. Topological data was collected using a 3-D optical profilometer providing valuable information about the surface roughness and heights at individual points.

These three datasets were spatially matched and imported into a graphical user interface, providing expert art conservators and historians with a comprehensive analytical tool to aid their endeavours in artwork conservation. The *Artwork Analysis Tool* was able to identify chemical and colour components of the painting sample complemented by the micro topological data. The Raman spectra were analysed using a dynamic Gaussian peak-fitting algorithm which highlighted the position and strength of detected Raman peaks. A Raman spectral database of common minerals, pigments, and binding media was used with the highlighted spectral peaks to identify chemical structures in the paintings. The reflectance spectra obtained were plotted in the GUI with a corresponding visual representation of the colour. This was in turn supplemented with topological information providing information about the reflectivity of the surface due to surface roughness and any surface damage. With Raman peaks highlighted and the corresponding colour illustrated for any point on the painting, the GUI can provide valuable insight for any user. The fusion of three different data modalities, which are typically used in isolation, was accomplished through the implementation of the *Artwork Analysis Tool*. The authors believe that the digital platform can improve the quality of the data obtained from the three techniques, heightening their potential for artwork analysis. As such, this tool can be used by art conservators and historians to gain precise information on the constituents of a painting for their endeavours in conserving precious works of art.

The *Artwork Analysis Tool* was a strong demonstration of how data from different modalities can be combined in a comprehensive manner to aid artwork conservators. However, there is an opportunity to discuss future work that intends to improve the existing system. Currently, the processing and preparation of the data require the running of a MATLAB script. This methodology is not ideal for those collecting the data to run in preparation for a session with the user. As such, a system that automates this data processing which is also easy for the typical user to instigate would improve the *Artwork Analysis Tool*. Data collected in near real-time by portable instruments could use this pipeline to produce results in the GUI as the analysis is being made. In addition, this would lead to the GUI accepting different types of data and intelligently determining how to represent the data. This would improve the versatility of the *Artwork Analysis Tool*, making it a better tool for expert artwork conservators and historians.

## Data Availability

Not applicable.

## References

[1] Calvini R., Ulrici A., Amigo J.M. (2020). Growing applications of hyperspectral and multispectral imaging. Data Handling in Science and Technology.

[2] Bin Han Y., Lee N.R., Kim Y.M., Shin J.A., Cha S.M., Kwon H.H. (2021). Paints analysis and conservation treatment of painted sculpture: Jean Dubuffet, Guard Dog II. SN Appl. Sci..

[3] Ciano C., Flammini M., Giliberti V., Calvani P., DelRe E., Talarico F., Torre M., Missori M., Ortolani M. (2018). Confocal Imaging at 0.3 THz With Depth Resolution of a Painted Wood Artwork for the Identification of Buried Thin Metal Foils. IEEE Trans. Terahertz Sci. Technol..

[4] Conti C., Colombo C., Realini M., Matousek P. (2015). Subsurface Analysis of Painted Sculptures and Plasters Using Mi-crometre-Scale Spatially Offset Raman Spectroscopy (Micro-Sors). J. Raman Spectrosc..

[5] Borg B., Dunn M., Ang A., Villis C. (2020). The Application of State-of-the-Art Technologies to Support Artwork Conser-vation: Literature Review. J. Cult. Herit..

[6] Artesani A., Ghirardello M., Mosca S., Nevin A., Valentini G., Comelli D. (2019). Combined photoluminescence and Raman microscopy for the identification of modern pigments: Explanatory examples on cross-sections from Russian avant-garde paintings. Heritage Sci..

[7] Invernizzi C., Fiocco G., Iwanicka M., Targowski P., Piccirillo A., Vagnini M., Licchelli M., Malagodi M., Bersani D. (2021). Surface and Interface Treatments on Wooden Artefacts: Potentialities and Limits of a Non-Invasive Multi-Technique Study. Coatings.

[8] Manfredda N., Buscaglia P., Gallo P., Borla M., Aicardi S., Poggi G., Baglioni P., Nervo M., Scalarone D., Borghi A. (2021). An Ancient Egyptian Multilayered Polychrome Wooden Sculpture Belonging to the Museo Egizio of Torino: Characterization of Painting Materials and Design of Cleaning Processes by Means of Highly Retentive Hydrogels. Coatings.

[9] Nardo V.M., Renda V., Anastasio G., Caponetti E., Saladino M., Vasi C., Ponterio R. (2019). A combination of portable non-invasive techniques to study on reverse glass paintings at Mistretta museum. Microchem. J..

[10] Pagliarulo V., Rippa M., Lanzillo A., Fatigati G., Rossi P., Grilli M., Mormile P., Ferraro P. Full-field NDT methods for investigation of paintings on poplar. Proceedings of the Multimodal Sensing and Artificial Intelligence: Technologies and Applications II SPIE.

[11] Daffara C., Mazzocato S., de Rubeis T., Ambrosini D. A simple method for artworks monitoring by simultaneous speckle interferometry (ESPI) and speckle photography. Proceedings of the Optics for Arts, Architecture, and Archaeology VIII SPIE.

[12] Degano I., Modugno F., Bonaduce I., Ribechini E., Colombini M.P. (2018). Recent Advances in Analytical Pyrolysis to In-vestigate Organic Materials in Heritage Science. Angew. Chem. Int. Ed..

[13] Fardi T., Pintus V., Kampasakali E., Pavlidou E., Schreiner M., Kyriacou G. (2018). Analytical characterization of artist’s paint systems based on emulsion polymers and synthetic organic pigments. J. Anal. Appl. Pyrolysis.

[14] Fontana D., Alberghina M.F., Barraco R., Basile S., Tranchina L., Brai M., Gueli A., Troja S.O. (2014). Historical pigments characterisation by quantitative X-ray fluorescence. J. Cult. Heritage.

[15] Buchta D., Hein N., Pedrini G., Krekel C., Osten W. Combination of topology and structural information for damages and deterioration analysis of artworks. Proceedings of the Optics for Arts, Architecture, and Archaeology V SPIE.

[16] Garciá-Molina D.F., López-Lago S., Hidalgo-Fernandez R.E., Triviño-Tarradas P. (2021). Digitalization and 3d Documentation Techniques Applied to Two Pieces of Visigothic Sculptural Heritage in Merida through Structured Light Scanning. J. Comput. Cult. Herit..

[17] Parraman C. The visual appearance and surface texture of materials according to the old masters. Proceedings of the Measuring, Modeling, and Reproducing Material Appearance.

[18] Fovo A.D., Tserevelakis G.J., Papanikolaou A., Zacharakis G., Fontana R. (2019). Combined photoacoustic imaging to delineate the internal structure of paintings. Opt. Lett..

[19] Targowski P., Góra M., Bajraszewski T., Szkulmowski M., Wojtkowski M., Kowalczyk A., Rouba B., Tymińska-Widmer L., Iwanicka M. Optical Coherence Tomography for Structural Imaging of Artworks. Proceedings of the Lasers in the Conservation of Artworks–Proceedings of the International Conference LACONA 7.

[20] Edwards H.G.M., Munshi T. (2005). Diagnostic Raman spectroscopy for the forensic detection of biomaterials and the preservation of cultural heritage. Anal. Bioanal. Chem..

[21] De Caro L., Matricciani E., Fanti G. (2018). Imaging Analysis and Digital Restoration of the Holy Face of Manoppello—Part II. Heritage.

[22] Aceto M., Arrais A., Marsano F., Agostino A., Fenoglio G., Idone A., Gulmini M. (2015). A diagnostic study on folium and orchil dyes with non-invasive and micro-destructive methods. Spectrochim. Acta Part A Mol. Biomol. Spectrosc..

[23] Fontana R., Fovo A.D., Striova J., Pezzati L., Pampaloni E., Raffaelli M., Barucci M. (2015). Application of non-invasive optical monitoring methodologies to follow and record painting cleaning processes. Appl. Phys. A.

[24] Mazzocato S., Daffara C. (2021). Experiencing the Untouchable: A Method for Scientific Exploration and Haptic Fruition of Artworks Microsurface Based on Optical Scanning Profilometry. Sensors.

[25] Mazzocato S., Marchioro G., Menegazzi A., Daffara C. Optical Micro-Profilometry for Surface Analysis and 3d Printed Replica of Archeological Artefacts. Proceedings of the 2020 IMEKO TC-4 International Conference on Metrology for Archaeology and Cultural Heritage.

[26] Cadusch P.J., Hlaing M.M., Wade S.A., McArthur S.L., Stoddart P.R. (2013). Improved methods for fluorescence background subtraction from Raman spectra. J. Raman Spectrosc..

[27] Burgio L., Clark R.J. (2001). Library of FT-Raman spectra of pigments, minerals, pigment media and varnishes, and supplement to existing library of Raman spectra of pigments with visible excitation. Spectrochim. Acta Part A Mol. Biomol. Spectrosc..

[28] Picollo M., Basilissi G., Cucci C., Stefani L., Tsukada M. Ultraviolet, Visible and Near Infrared Reflectance Spectra of Modern Pictorial Materials in the 200–2500 nm Range. https://spectradb.ifac.cnr.it/drs/.

